# Knee extensor force production and discomfort during neuromuscular electrical stimulation of quadriceps with and without gluteal muscle co-stimulation

**DOI:** 10.1007/s00421-022-04949-9

**Published:** 2022-04-15

**Authors:** J. Flodin, C. Mikkelsen, P. W. Ackermann

**Affiliations:** 1 Integrative Orthopedic Laboratory, Department of Molecular Medicine and Surgery, Karolinska Institutet, Stockholm, Sweden; 2 Department of Trauma, Acute Surgery and Orthopaedics, Karolinska University Hospital, 171 76 Stockholm, Sweden; 3 Stockholm Sports Trauma Research Center, Department of Molecular Medicine and Surgery, Karolinska Institutet, Stockholm, Sweden; 4 Capio Artro Clinic, Stockholm, Sweden

**Keywords:** Electrical stimulation therapy, Patient comfort, Pain, Muscle stimulation, Skeletal muscles

## Abstract

**Purpose:**

To investigate whether Neuromuscular Electrical Stimulation (NMES) simultaneously applied on the quadriceps (Q) and gluteal (G) muscles, as compared to single Q-stimulation alters the knee extensor force production and discomfort.

**Methods:**

A total of 11 healthy participants (6 females), with normal weight and age between 19 and 54 years were included. The unilateral, isometric maximal voluntary contraction (MVC) was assessed for each participant in an isokinetic dynamometer (Biodex, system 3). NMES was, in a randomized order, applied only on the Q-muscle and on the Q- and G-muscles (QG) simultaneously. NMES-intensity was increased stepwise until the maximal tolerable level was reached regarding discomfort, graded according to the visual analogue scale (VAS). VAS and the % of MVC produced by NMES, were registered for each level, expressed as median (inter-quartile range).

**Results:**

The maximum tolerated NMES-intensity applied on Q compared to QG resulted in equally high discomfort, 8.0 (6.0–9.0) vs 8.0 (6.3–9.0), and in equivalent knee extensor force production, 36.7 (29.9–47.5) and 36.2 (28.9–49.3), respectively, in % of MVC. At 20% of MVC, NMES applied on Q compared to QG resulted in equal acceptable discomfort, 3.0 (2.0–4.5) vs 3.0 (3–5.5), and comparable intensity levels, 41.5 (38.0–45.8) vs 43.5 (37.0–48.8), respectively.

**Conclusions:**

Simultaneous QG-NMES, as compared to single Q-NMES, does not seem to affect the knee extensor force production or discomfort. Q-NMES, without voluntary muscle contraction, can with an acceptable level of discomfort result in at least 20% of MVC.

## Introduction

Periods of inactivity or immobilization, for example after an injury or disease, lead to loss of muscle strength and mass (Rubenstein 2006). One way to prevent this is to exercise the quadriceps (Q) and gluteal (G) muscles, which are crucial for postural stability and gait (Ahmadiahangar et al. 2018; Inacio et al. 2014). An alternative way to prevent loss of muscle strength in involuntary inactive persons, such as after surgery or due to advanced disease, is to activate the muscles using neuromuscular electrical stimulation (NMES) (Caulfield et al. 2013; Langeard et al. 2017; Snyder-Mackler et al. 1994). The drawback of current NMES-application is, however, that many patients experience discomfort, leading to low compliance to the treatment (Doheny et al. 2010; Glaviano and Saliba 2016; Lyons et al. 2004).

NMES for muscle strengthening purposes has mostly been studied together with voluntary muscle co-contractions (Benavent-Caballer et al. 2014; Langeard et al. 2017), which is not always possible for injured and immobilized patients. Thus, some studies have investigated which degree of maximal voluntary contraction (MVC) patients can attain when using Q-NMES without voluntary muscle contraction (Glaviano and Saliba 2016; Langeard et al. 2017; Vivodtzev et al. 2012). There is, however, no consensus on the % of MVC that can be reached with an acceptable level of discomfort (e.g., VAS below 4) (Myles et al. 2017).

Moreover, in immobile patients unable to produce voluntary muscle contractions it may be beneficial to activate several muscles (Duignan et al. 2019), such as the Q- and G-muscles, which imitates the physiological muscle actions during functional activities. QG co-stimulation may save time for both personnel and patients and result in enhanced rehabilitation effects on, e.g., postural stability (Ahmadiahangar et al. 2018; Inacio et al. 2014). However, whether the Q- and G-muscles can be treated with NMES simultaneously without increasing the discomfort, or if the G co-stimulation may affect the knee extensor force production in either a negative or a positive way is unknown.

This explorative study on healthy participants was conducted to improve the NMES-application in immobilized patients unable to perform voluntary contractions. The aim of this study was to assess the knee extensor force production and participant discomfort, without voluntary muscle contractions, during increasing NMES-intensities applied on Q alone or QG together. A secondary aim was to verify if the submaximal torque level of 20% MVC, which corresponds to the lower range needed for strengthening effects, could be attained with an acceptable level of discomfort. We hypothesized that the knee extensor force during Q-NMES would increase during co-stimulation of G-NMES, while the participant discomfort would be unaffected. A second hypothesis was that the knee extensor force as well as discomfort would be linearly proportional to the NMES-intensity applied on Q, independently of G co-stimulation.

## Methods

### Ethical approval

The study was performed in line with the principles of the Declaration of Helsinki. Ethical approval was obtained from the Regional Ethical Review Committees (Dnr: 2019–04,020) and (Dnr: 2021–05,076).

### Participants

A total of 11 healthy participants aged between 19 and 54 years participated in the study (Table 1). All participants completed a questionnaire about basic characteristics, were measured in height and weight and the estimated body fat for each participant was calculated using the US navy method (Peterson 2015). All participants reported that their right leg was the dominant one. All participants signed an informed consent before the study confirming that they did not meet any of the exclusion criteria. Exclusion criteria were pregnancy, skin ulcer, pacemaker, intracardiac defibrillator, advanced heart disease, kidney failure, neuromuscular or metabolic disease.

**Table 1:** Demographics and characteristics of the participants

Participant	Sex	Age, (Years)	Height, (cm)	Weight, (cm)	BMI, (kg/cm^2^)	Maximal Voluntary contraction (MVC), Nm	Physical activity level^a^	Body fat US navy method, (%)
1	Male	24	183	82	24.5	206	5	19.5
2	Female	25	171	66	22.6	128	6	23.4
3	Male	52	183	80	23.9	239	6	20.7
4	Female	54	172	66	22.3	99.4	5	27.8
5	Female	25	173	60	20.0	95.4	3	19.6
6	Male	24	186	82	23.7	216	6	9.40
7	Male	38	180	85	26.2	293	5	22.6
8	Female	54	174	70	23.1	108	3	31.2
9	Female	24	165	53	19.4	118	5	17.6
10	Female	34	169	60	21.0	134	6	21.1
11	Male	19	170	70	24.2	151	6	16.2
		25 (24–45)^b^	173 (171–182) ^b^	70 (63–81)^b^	23.1 (21.6–24.1)^b^	134 (113–211) ^b^	5 (5–6)^b^	20.7 (18.6–23)^b^

^a^Frändin/Grimby activity scale (1–6)

^b^Median (inter-quartile range)

### Physical activity level

The physical activity level was estimated using Frändin/Grimby activity scale, scored from 1 (no physical activity) to 6 (heavy physical exercise several times/week) (Frändin and Grimby 1994).

### Body fat US navy method

The estimated body fat for each participant was calculated using the US navy method (Peterson 2015), a reliable method for estimating body fat. The body fat was calculated based on a formula including the participants height, circumference of the neck, waist and hips (only women) (Peterson 2015).

### Maximal voluntary contraction (MVC) torque assessment

Isometric knee extension torque was measured using an isokinetic dynamometer (Biodex System 3 Pro; Biodex Medical Systems, Shirley, New York) with the knee joint in 90 degrees of flexion. The lateral epicondyle of the femur was aligned with the rotational axis of the dynamometer, and the back was reclined at approximately 100 degrees. Stabilizing belts were positioned across the chest, hips and thighs. The participants were instructed to place their arms on their chest. A well-trained physiotherapist supervised all the measurements.

Initially, the participant warmed up on a stationary bike for a duration of 5 min. Then the participant was positioned in the dynamometer as describe above. The leg used for the test was randomized based on the participant’s dominant/non-dominant leg. Thereafter the participants tested the isometric measurement in the dynamometer three times at submaximal levels. Thereafter participants completed 3 maximal isometric contractions of one leg for 6 s with the knee stationary at 90 degrees flexion. Each maximal isometric repetition was followed by a 30 s rest. During voluntary contractions, participants were encouraged verbally and received visual feedback during each repetition. The greatest peak torque achieved was used as the Maximal Voluntary contraction (MVC) torque (Newton meters, Nm) for further analysis. After the voluntary contraction, the tests with the NMES started.

### NMES procedures

#### Electrodes and placement

The electrode placement and size were based on a previous study comparing different pre-determined electrode configurations (Flodin et al. 2022). Self-adhesive electrodes (Compex Snap, Performance, DJO Global, USA) were used to apply the NMES. Two electrodes sized 5 × 5 cm (distal) and one electrode sized 5 × 9 cm (proximal) were placed over the Q-muscle while two electrodes sized 5 × 9 cm were used on the G-muscle. The skin was not shaved prior to the testing and the skin on the leg used for the test was washed with water and thoroughly dried with a towel before the start of the test. The stimulator was connected to two 1.5 m long isolated cables, and each cable was connected to two self-adhesive electrodes.

To determine the Q-placement of the electrodes on each participant, anatomical landmarks were marked out at the upper border of the patella and also at the point where the line between the left and right spina iliaca anterior superior (SIAS) intersected the line running from the upper border of the patella, parallel to the longitudinal midline of the participant´s body. The straight line connecting these two anatomical landmarks was defined as the “anterior mid-thigh line” (AMTL) and the length of the thigh was defined as the length of AMTL. Then, the circumference of the thigh was measured at 15% of the length of the thigh in proximal direction from the patella. The following electrode placements in medial and lateral direction from the AMTL was based on this circumference regardless of where on the thigh the electrodes were placed. The distal electrodes were placed at a point 90% (medial), respectively, and 80% (lateral) of the length of the AMTL distal to SIAS. The medial and lateral distance of electrodes from AMTL was 25% of the circumference. The proximal electrode was placed at a point 30% distal to SIAS along the AMTL.

To determine the G-placement of the electrodes, anatomical landmarks were marked out at the middle of the gluteal fold and also at the point where a line between the left and right iliac crest intersected the line running from the middle of the gluteal crest, parallel to the longitudinal midline of the participant´s body. The length of this line was measured and electrodes were placed at points 20% and 80% distal to the iliac crest on this line.

#### NMES-settings

Electrical stimulation was delivered through a constant-current NMES-device, Chattanooga Physio (DJO Nordic, Malmoe, Sweden). The waveform of the current delivered was a symmetrical, biphasic, square-form pulse, using a frequency of 36 Hz, a pulse duration of 800 µs (phase duration of 400 µs), contraction time of a total of 6 s (1 s ramp-up/-down time) and 12 s rest in between. A burst stimulation with the frequency of 1 Hz and an intensity of 5.5 mA was delivered during the rest-period. These settings were chosen based on previous studies (Doucet et al. 2012; Glaviano and Saliba 2016) as well as our own pilot experiments demonstrating optimal patient compliance. The intensity (amplitude) was gradually increased on the NMES-device (0–999), which represented a non-linear scale of current ranging from 0 to 120 milliampere (mA). The NMES-level needed for each test was translated to mA based on information from DJO Global.

### Experimental procedure

**Fig. 1:**
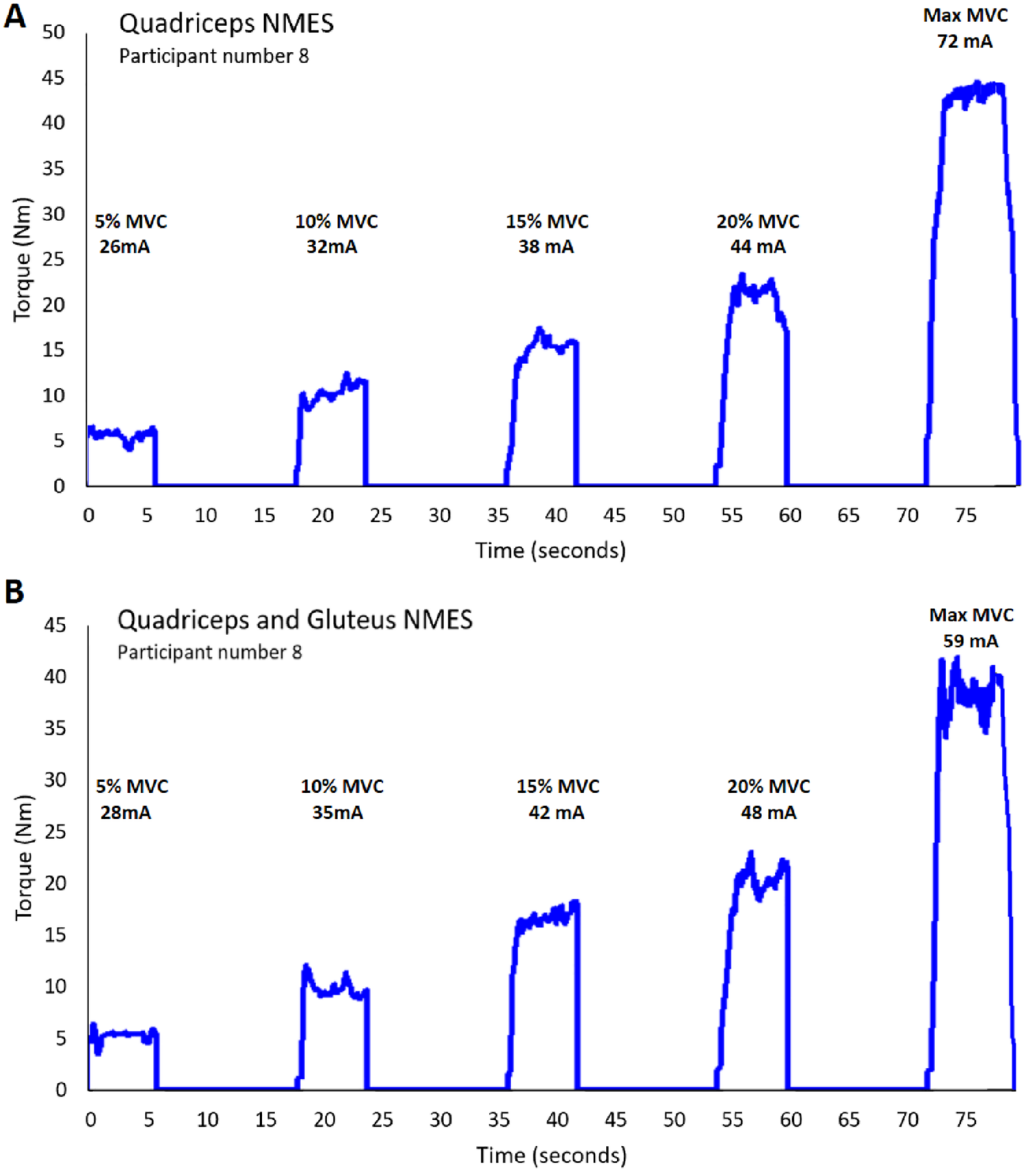
Example of real torque traces collected during quadriceps NMES (**A**) and quadriceps and gluteus NMES (**B**) from participant number 8 to illustrate how the data was collected. Each curve shows the torque in Nm produced during NMES at a level equal to 5, 10, 15 and 20% of MVC torque, as well as maximal MVC torque produced, and the corresponding NMES-intensity in mA needed. *MVC* Maximal Voluntary Contraction, *mA* milliAmpere, *NMES* Neuromuscular Electrical Stimulation, *Nm* Newton meter

Two separate conditions were performed for each participant. In one condition only the Q-muscle was stimulated with NMES and in the second condition both the Q- and G-muscles were stimulated simultaneously. The conditions were performed in a randomized order to minimize the effect of test order on the results and separated by 5 min rest between the two conditions. The NMES testing began after the participants had performed the warm-up and testing of MVC torque as described above. The intensity of the NMES was increased stepwise by initially 5 NMES-levels per stimulation and after an NMES-level of 40 by 10 NMES-levels per stimulation. For the participants that tolerated an NMES-level of 120 or above, the intensity was increased based on the participants’ discomfort at NMES-level 120 according to the following: VAS 0–3, 30 NMES-levels/stimulation, VAS 4–6, 20 NMES-levels/stimulation and VAS 7–10, 10 NMES-levels/stimulation. The intensity of NMES was increased synchronously on the Q- and G-muscles during the QG-stimulation. An example of real torque traces collected during Q- and QG-NMES from participant number 8 are displayed in Fig. 1 to illustrate how the data was collected.

#### Assessment of discomfort

At each NMES-level the participants were asked to evaluate the general discomfort during the stimulation according to the Visual Analogue Scale (VAS) on a 100-step scale (0–10, with one decimal (cm)), with 0 indicating no discomfort or pain and 10 indicating the worst imaginable discomfort or pain (Price et al. 1983). All participants were informed that they could end the stimulation whenever they wanted, but were encouraged to increase the intensity as much as tolerable.

#### Outcome measurements

The primary outcome measurement was the knee extensor force production during Q- vs QG-stimulation. Only the knee extensor force was measured in the dynamometer since it was not possible to measure a movement in any limb from the G-stimulation, but a clear tension in the G-muscle was evoked by the NMES. The knee extensor force produced by NMES was measured in the dynamometer (Nm). This value was then divided with the participant´s voluntary MVC torque, and expressed as % of MVC torque which was used as the primary outcome variable. Both the maximal (highest tolerable NMES-intensity) and submaximal level were assessed. The submaximal level (approximately 20% of MVC torque) was chosen based on the % of MVC torque the participants could reach below VAS 4, since this was considered as an acceptable level of discomfort (Myles et al. 2017). Moreover, previous studies have indicated that 20% of MVC torque corresponds to the lower range of force production that is needed for muscle strengthening effects (Maffiuletti 2010; Rabello et al. 2021). The secondary outcomes were the discomfort (according to VAS) and % of MVC torque at different NMES-intensities, the highest NMES-intensity the participants could tolerate below VAS 4 as well as the inter-individual variation between participants.

### Statistical analysis

The data were analyzed using SPSS version 25 (IBM Corp. Released 2016. IBM SPSS Statistics for Windows, Armonk, NY: IBM Corp.) in cooperation with a statistician. The sample size was calculated on 10 mA expected difference between Q- and QG-stimulation. Eight patients were required to detect a difference of 10 mA in NMES (two-sided type-I error rate = 5%; power = 80%), and a total of eleven patients were included. All variables were checked for skewness with Shapiro–Wilks test and most of the variables were normally distributed. However, due to the low number of participants (*n* = 11), all variables (patient characteristics, comfort according to VAS and required intensity at submaximal, as well as highest tolerated intensity and produced % of MVC torque at maximal level) were summarized with descriptive statistics such as median, inter-quartile range and frequency and the Wilcoxon signed rank test was used for the inferential statistics.

## Results

### Maximum knee extensor force production by Q- and QG-NMES

At the maximum level of tolerance, NMES applied on the Q-muscle compared to both the Q- and G-muscles (QG) resulted in a median knee extensor force production of 36.7% (range 28.2–58.8%) and 36.2% (20.9–60.6%), respectively, of MVC torque (*p* = 0.722) (Table 2). The discomfort assessed by the participants according to VAS at the maximum level of tolerance was high, with a median VAS 8 for both QG- and Q-stimulation (*p* = 0.131). The NMES intensity required to reach the maximum level of tolerance was slightly lower with QG-stimulation, median mA (range), 53.5 (38–131) compared to Q-stimulation 58.5 (36–125); however, the difference was not significant (*p* = 0.197) (Table 2).

**Table 2:** Maximum % of MVC torque produced by NMES, maximum intensity tolerated and participant discomfort at the maximum level on quadriceps (Q) only, respectively, quadriceps and gluteus (QG) muscles

Participant	Max% MVC torque		mA		VAS	
	Q	QG	Q	QG	Q	QG
1	48.6	52.5	83.5	69.0	8	9
2	31.2	37.4	53.5	49.0	6	6
3	30.1	30.5	80.0	70.5	9	9
4	28.2	27.2	50.5	49.0	9	9
5	36.7	20.9	53.5	49.0	9	10
6	58.8	60.6	125	131	10	10
7	29.0	25.6	66.0	53.5	6	6
8	40.9	36.2	72.0	58.5	5	5
9	29.6	35.6	36.0	38.0	6	7
10	46.4	46.0	53.5	46.5	7	7
11	50.4	53.7	58.5	62.5	9	8.5
Median (Inter-quartile range)	36.7% (29.9–47.5)	36.2% (28.9–49.3)	58.5 (53.5–76)	53.5 (49.0–65.8)	8.0 (6.0–9.0)	8.0 (6.3–9.0)

*Q* Quadriceps muscle, *QG* Quadriceps and Gluteus muscles, *mA*  milliAmpere, *VAS* Visual Analog Scale, *MVC* Maximal voluntary contraction

### Submaximal knee extensor force production by Q- and QG-NMES

At the submaximal level of NMES, when the torque production in knee extension reached 20% of MVC torque, the discomfort of the participants according to VAS was acceptable, median (range) of 3 (0–8) for Q-stimulation and 3 (1–10) for QG-stimulation (*p* = 0.179). The median mA required to reach the submaximal level was similar for QG-stimulation, 43.5 mA compared to Q-stimulation, 41.5 mA (*p* = 0.959) (Table 3).

**Table 3:** Intensity and participant discomfort at a submaximal level (approximately 20% of MVC torque) produced by only NMES on quadriceps (Q), respectively, quadriceps and gluteus (QG) muscles

Participant	% MVC torque		mA		VAS	
	Q	QG	Q	QG	Q	QG
1	20.9	20.9	43.5	41.5	0	3
2	21.8	21.8	40.0	36.0	2	3
3	20.1	19.2	63.0	52.0	5	7
4	20.1	21.1	41.5	43.5	8	7
5	18.3	18.9	41.5	48.5	5	10
6	19.9	19.9	41.5	50.5	3	4
7	19.1	19.1	49.0	45.5	3	3
8	22.3	21.8	48.0	49.0	1	2
9	21.2	21.2	29.5	29.5	4	3
10	22.5	22.1	36.0	34.0	2	4
11	19.9	20.6	31.5	38.0	4	1
Median (Inter-quartile range)	20.1% (19.9–21.5)	20.9% (19.6–21.5)	41.5 (38.0–45.8)	43.5 (37.0–48.8)	3.0 (2.0–4.5)	3.0 (3.0–5.5)

*Q* Quadriceps muscle, *QG* Quadriceps and Gluteus muscles, *mA* milliAmpere, *VAS* Visual Analoge Scale, *MVC* Maximal voluntary contraction

### Knee extensor force production in relation to NMES intensity

**Fig. 2:**
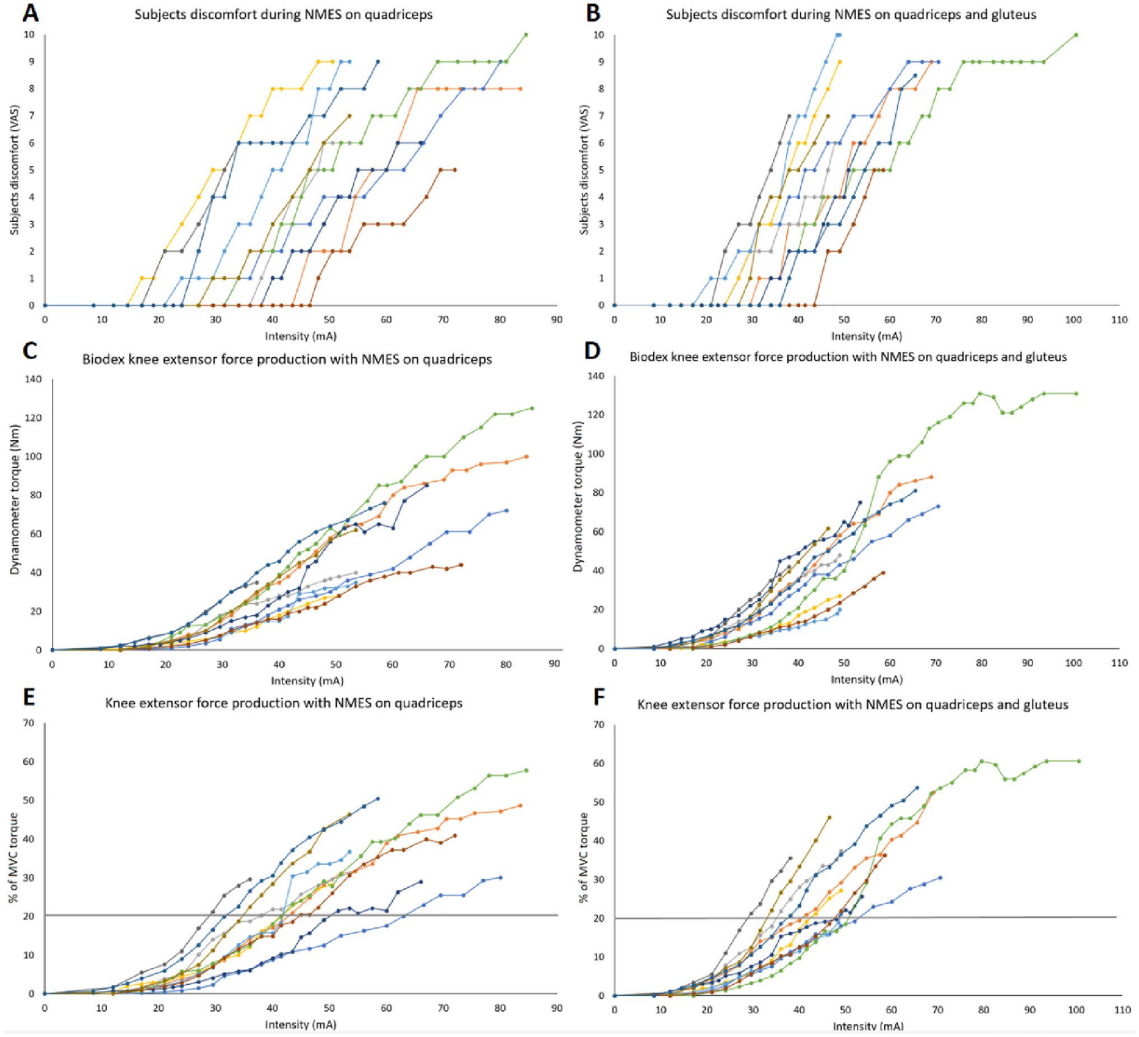
Each individuals discomfort estimated by the participants according to VAS (**A**, **B**) and knee extensor force production measured in Nm (**C**, **D**) and % of MVC torque (**E**, **F**) for different NMES intensities (mA) when stimulating the quadriceps muscles (**A**, **C**, **E**) and the quadriceps and the gluteus muscles simultaneously (**B**, **D**, **F**). Each different colored curve represents one participant. The horizontal lines represent the submaximal level of 20% of MVC torque (**E**, **F**). *MVC* Maximal Voluntary Contraction, *mA*  milliAmpere, *NMES* Neuromuscular Electrical Stimulation, *Nm* Newton meter

The inter-individual variation in knee extensor force production (measured both in Nm and % of MVC torque) was generally high and increased as the NMES-intensity (mA) was amplified. This finding was similar for Q-stimulation and QG-stimulation (Fig. 2C–F). In this study the inter-individual variation in NMES-intensity required to produce a certain % of MVC torque varied around twofold (e.g., 30–60 mA for 20%MVC and 40–80 mA for 30%MVC). The maximum tolerable % of MVC torque attained varied between around 30% up to 60% of MVC torque.

**Fig. 3:**
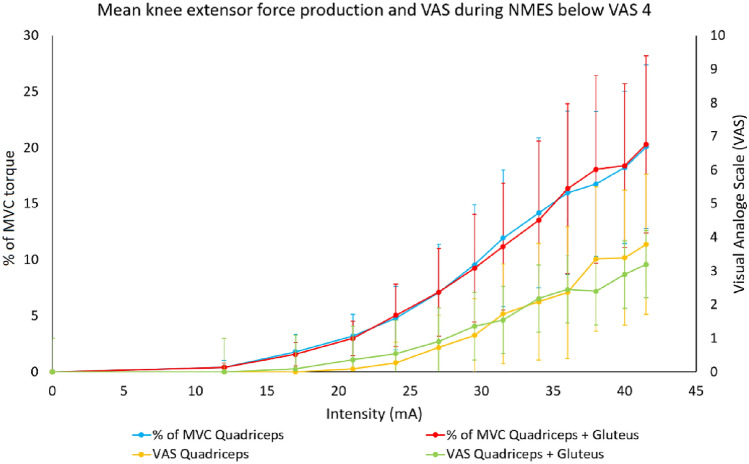
Mean knee extensor force production (% of MVC torque) and mean discomfort estimated by the participants according to VAS for different NMES intensities (mA) below VAS 4 when stimulating the quadriceps muscles, and the quadriceps and the gluteus muscles simultaneously. *MVC* Maximal Voluntary Contraction, *mA* milliAmpere, *NMES* Neuromuscular Electrical Stimulation

Each participant’s knee extensor force curve exhibited a relatively low, non-linear, increase in % of MVC torque from 0 mA up to 20–30 mA. Subsequently each curve exhibited a higher and more linear increase in % of MVC torque per increase of NMES intensity between 25 and 45 mA, corresponding to 5–20% of MVC torque (Fig. 3). Similar curves were observed for Q-stimulation as compared to QG-stimulation (Fig. 2C–F).

### Discomfort in relation to NMES intensity

The inter-individual variation in discomfort according to VAS was overall high but increased at approximately similar rates for the different participants as the NMES-intensity (mA) was increased. This observation was similar for both Q- and QG-stimulation (Fig. 2A, B). In this cohort the inter-individual variation in NMES-intensity at distinct VAS-levels varied approximately 30 mA (e.g., 15–45 mA at VAS 1, 25–55 mA at VAS 3 and 35–65 mA at VAS 6).

The VAS at the maximum level of tolerance of the participants varied between VAS 5–10, for both Q- and QG-stimulation (Fig. 2A, B). The mean curve seemed to exhibit a linear relationship between current intensity and VAS experience between an intensity of approximately 25 mA to 45 mA (Fig. 3). However, while all participants demonstrated approximately parallel curves, their starting points (e.g., the intensity the participants first reported anything but VAS 0) were dispersed with intensities between 10 and 45 mA (Fig. 2A, B).

## Discussion

In this study we found that the knee extensor force production and participant discomfort during NMES on the Q-muscle were not affected by simultaneous NMES of the G-muscle. With an NMES-intensity equal to the maximum level of tolerance, participants could reach around one third of the knee extensor force as compared to the participants own maximal voluntary contraction (MVC) torque, without a voluntary co-contraction, both when stimulating on the Q-muscle solely and on Q- and G-muscles (QG) simultaneously. Another main finding of the study was that NMES-treatment corresponding to a knee extension force of 20% of MVC torque (the submaximal level) resulted in an acceptable discomfort for the participants.

The main finding of our study, which to the best of our knowledge has not been published before, was that it is possible to apply NMES to both the Q- and G-muscles simultaneously without increasing the discomfort of the participant. In addition, QG-stimulation did not produce a superior knee extension force as compared to solely Q-stimulation. This was expected, since the Q-muscle is the only muscle contributing to the force in voluntary knee extension (Alkner et al. 2000), but has to the best of our knowledge not been demonstrated before. However, the Q- and G-muscles are connected since one of the Q-muscles, i.e., rectus femoris, contributes to hip flexion, in addition to knee extension, and, therefore, works as an antagonist to G-muscle (Thompson 2014). Hypothetically, a stabilization of hip flexion, by coactivation of agonist and antagonist, could enhance the strength of rectus femoris in knee extension. However, this concept was not demonstrated in this study. One previous study has found that co-stimulation of the Q- and hamstrings muscles, which are antagonist in knee extension, is possible without increasing the discomfort or maximum produced knee extensor force (Duignan et al. 2019), which are in line with our findings during co-stimulation on the Q- and G-muscles. When applying NMES on immobilized patients, such combined stimulation of inactive muscle groups could be of importance for enhanced rehabilitation effects (Duignan et al. 2019).

For five of the eleven participants in this study, however, NMES co-stimulation of the Q and G-muscles resulted in a higher maximal knee extensor force production (3–15% higher % of MVC torque). This finding may be explained by the observation that the discomfort decreased for the participants when both muscle groups were stimulated simultaneously. This is in line with the concept of “pain inhibits pain”, or gate-control theory (Yarnitsky 2010), and could be an explanation for the observation that some of the participants tolerated higher NMES-intensity during co-stimulation of the two muscles and, therefore, also increased their maximum knee extensor force. In addition, inter-joint coupling effects can contribute to the force production during muscle co-activation (Fox et al. 2020; Antwerp et al. 2007), which also could be an explanatory factor to the increased force production during QG-stimulation. On the other hand, four other participants expressed that they experienced more discomfort during muscle co-stimulation. It was observed in these four participants that the same NMES-intensity was more painful in the G-area than in the Q-area, which resulted in earlier termination of the QG stimulation. In future studies it would be interesting to investigate if the discomfort could be decreased if the intensity was increased separately for the two muscle groups, so that the second muscle group experienced a non-painful sensation as stated in the gate control theory.

A second main finding of this study was that at the submaximal level, the participants experienced a median VAS of 3, which is considered as an acceptable level of discomfort during treatment (Myles et al. 2017). This observation, in combination with the finding that all study participants were able to reach the submaximal level, suggests that this level may be suitable for further studies on NMES muscle strengthening effects in immobilized patients without the ability to perform voluntary muscle contractions. This conclusion is supported by some previous studies indicating that 20% of MVC torque corresponds to the lower range of force production that is needed for muscle strengthening effects (Maffiuletti 2010; Rabello et al. 2021). However, since NMES preferentially targets fast motor units and recruits motor units in a non-selective manner (Glaviano and Saliba 2016), it is possible that 20% of MVC torque induced by the NMES represents a higher degree of muscle activation as compared to 20% MVC torque with voluntary muscle contraction. This would seem to represent that NMES is beneficial for fast-twitch muscle fibers, which are often affected after injury and surgery (Lievens et al. 1985), corroborating the notion that an NMES-level that will induce a force equivalent to 20% of MVC torque may be effective for Q strengthening in hospitalized and immobilized patients (Maffiuletti 2010). This study also found that participants displayed an approximately linear increase in % of MVC torque and VAS between an intensity of 25–45 mA which equals to 5–20% of MVC torque. However, at lower intensities the increase was not linear and a possible explanation to this is that a certain intensity is needed to reach the sensory and motor threshold, and that the intensity needed for this differ among different individuals (Maffiuletti et al. 2008).

A third main finding of this study was the huge inter-individual variation in NMES-intensity required to reach a specific knee extensor force production % of MVC torque, which is in line with previous research (Maffiuletti et al. 2011; Medeiros et al. 2015). The differences in intensity required increased as force production was augmented. Important contributing factors to this variation in intensity requirement have been found in the participants´ body composition (Doheny et al. 2010; Maffiuletti et al. 2011; Medeiros et al. 2015), including subcutaneous fat layer thickness (Maffiuletti et al. 2011; Medeiros et al. 2015), weight and BMI (Doheny et al. 2010; Maffiuletti et al. 2011), and sex (Maffiuletti et al. 2008, 2011). Our study only included healthy participants with normal BMI who exercised regularly, while the population in the previous studies (Doheny et al. 2010; Maffiuletti et al. 2011; Medeiros et al. 2015) varied highly regarding BMI of the participants. Therefore, a population with more variation in BMI would, based on previous studies (Doheny et al. 2010; Maffiuletti et al. 2011; Medeiros et al. 2015), result in larger variations in intensity needed for muscle contraction than we found in this study. In this study the sample size was too small to investigate the effect of participant characteristics. However, this would be an interesting aspect to investigate further in future studies.

One possible limitation in this study was that the sample size was quite small (*n* = 11) and only included healthy participants which may not be representative for the patient population NMES-treatment is aiming towards in the end. However, this was an explorative study with the aim to investigate if it was possible to stimulate Q- and G-muscle simultaneously to perform future studies on immobilized patients. Another possible limitation is that the testing of Q- compared to QG-stimulation was performed during the same session with 5 min rest in-between. This may result in possible adaptation to the sensation of NMES in the second condition as well as development of more fatigue in the second condition, based on previous studies (Doucet et al. 2012; Karlsen et al. 2020; Maffiuletti 2010). However, to control for potential adaptation to the sensation as well as possible higher fatigue for the second condition, we randomized the order of the two conditions. In addition, it is possible that participants experienced some fatigue during the increasing stimulation intensity at each condition. However, the linear form of the curves of current intensity vs % of MVC torque reflects few signs of fatigue.

## Conclusions

In conclusion, we found that during quadriceps NMES, simultaneous gluteus NMES did not affect the knee extensor force production or participant discomfort. Quadriceps NMES, without voluntary co-contraction, can with an acceptable level of discomfort produce at least 20% of MVC torque. These results provide a foundation for further studies of individualized NMES-settings for muscle strength and mass preservation in immobilized patients without voluntary contraction.

## Data Availability

The data generated and/or analyzed during the current study are not publicly available but are available from the corresponding author on reasonable request.

## Abbreviations

AMTL: Anterior mid-thigh line
G: Gluteus
BMI: Body Mass Index
MVC: Maximal voluntary contraction
mA: MilliAmpere
NMES: Neuromuscular electrical stimulation
Nm: Newton meters
SIAS: Spina iliaca anterior superior
VAS: Visual Analogue scale
QG: Quadriceps- and gluteus
Q: Quadriceps

## References

[CR1] Ahmadiahangar A, Javadian Y, Babaei M (2018). The role of quadriceps muscle strength in the development of falls in the elderly people, a cross-sectional study. Chiropr Man Therap.

[CR2] Alkner BA, Tesch PA, Berg HE (2000). Quadriceps EMG/force relationship in knee extension and leg press. Med Sci Sports Exerc.

[CR3] Benavent-Caballer V, Rosado-Calatayud P, Segura-Orti E, Amer-Cuenca JJ, Lison JF (2014). Effects of three different low-intensity exercise interventions on physical performance, muscle CSA and activities of daily living: a randomized controlled trial. Exp Gerontol.

[CR4] Caulfield B, Prendergast A, Rainsford G, Minogue C (2013). Self directed home based electrical muscle stimulation training improves exercise tolerance and strength in healthy elderly. Conf Proc IEEE Eng Med Biol Soc.

[CR5] Doheny EP, Caulfield BM, Minogue CM, Lowery MM (2010). Effect of subcutaneous fat thickness and surface electrode configuration during neuromuscular electrical stimulation. Med Eng Phys.

[CR6] Doucet BM, Lam A, Griffin L (2012). Neuromuscular electrical stimulation for skeletal muscle function. Yale J Biol Med.

[CR7] Duignan C, Doolan M, Doyle D (2019). A performance comparison of neuromuscular electrical stimulation protocols for isolated quadriceps contraction versus co-contraction of quadriceps and hamstrings. Annu Int Conf IEEE Eng Med Biol Soc.

[CR8] Flodin J, Juthberg R, Ackermann PW (2022). Effects of electrode size and placement on comfort and efficiency during low-intensity neuromuscular electrical stimulation of quadriceps, hamstrings and gluteal muscles. BMC Sports Sci Med Rehabil.

[CR9] Fox JW, Jagodinsky AE, Wilburn CM, Smallwood L, Weimar WH (2020). Lower extremity joints and their contributions to whole limb extension. Int Biomech.

[CR10] Frändin K, Grimby G (1994). Assessment of physical activity, fitness and performance in 76-year-olds. Scand J Med Sci Sports.

[CR11] Glaviano NR, Saliba S (2016). Can the use of neuromuscular electrical stimulation be improved to optimize quadriceps strengthening?. Sports Health.

[CR12] Inacio M, Ryan AS, Bair WN (2014). Gluteal muscle composition differentiates fallers from non-fallers in community dwelling older adults. BMC Geriatr.

[CR13] Karlsen A, Cullum CK, Norheim KL (2020). Neuromuscular electrical stimulation preserves leg lean mass in geriatric patients. Med Sci Sports Exerc.

[CR14] Langeard A, Bigot L, Chastan N, Gauthier A (2017). Does neuromuscular electrical stimulation training of the lower limb have functional effects on the elderly?: A systematic review. Exp Gerontol.

[CR15] Lievens E, Klass M, Bex T, Derave W (2020). Muscle fiber typology substantially influences time to recover from high-intensity exercise. J Appl Physiol (1985).

[CR16] Lyons GM, Leane GE, Clarke-Moloney M, O'Brien JV, Grace PA (2004). An investigation of the effect of electrode size and electrode location on comfort during stimulation of the gastrocnemius muscle. Med Eng Phys.

[CR17] Maffiuletti NA (2010). Physiological and methodological considerations for the use of neuromuscular electrical stimulation. Eur J Appl Physiol.

[CR18] Maffiuletti NA, Herrero AJ, Jubeau M, Impellizzeri FM, Bizzini M (2008). Differences in electrical stimulation thresholds between men and women. Ann Neurol.

[CR19] Maffiuletti NA, Morelli A, Martin A (2011). Effect of gender and obesity on electrical current thresholds. Muscle Nerve.

[CR20] Medeiros FV, Vieira A, Carregaro RL (2015). Skinfold thickness affects the isometric knee extension torque evoked by Neuromuscular Electrical Stimulation. Braz J Phys Ther.

[CR21] Myles PS, Myles DB, Galagher W (2017). Measuring acute postoperative pain using the visual analog scale: the minimal clinically important difference and patient acceptable symptom state. Br J Anaesth.

[CR22] Peterson DD (2015). History of the U.S. Navy Body Composition program. Mil Med.

[CR23] Price DD, McGrath PA, Rafii A, Buckingham B (1983). The validation of visual analogue scales as ratio scale measures for chronic and experimental pain. Pain.

[CR24] Rabello R, Fröhlich M, Maffiuletti NA, Vaz MA (2021). Influence of pulse waveform and frequency on evoked torque, stimulation efficiency, and discomfort in healthy subjects. Am J Phys Med Rehabil.

[CR25] Rubenstein LZ (2006). Falls in older people: epidemiology, risk factors and strategies for prevention. Age Ageing.

[CR26] Snyder-Mackler L, Delitto A, Stralka SW, Bailey SL (1994). Use of electrical stimulation to enhance recovery of quadriceps femoris muscle force production in patients following anterior cruciate ligament reconstruction. Phys Ther.

[CR27] Thompson M (2014). The contribution of the rectus femoris to hip flexion. J Athl Enhanc.

[CR28] van Antwerp KW, Burkholder TJ, Ting LH (2007). Inter-joint coupling effects on muscle contributions to endpoint force and acceleration in a musculoskeletal model of the cat hindlimb. J Biomech.

[CR29] Vivodtzev I, Debigaré R, Gagnon P (2012). Functional and muscular effects of neuromuscular electrical stimulation in patients with severe COPD: a randomized clinical trial. Chest.

[CR30] Yarnitsky D (2010). Conditioned pain modulation (the diffuse noxious inhibitory control-like effect): its relevance for acute and chronic pain states. Curr Opin Anaesthesiol.

